# Dose-Dependent Cytotoxicity of Polypropylene Microplastics (PP-MPs) in Two Freshwater Fishes

**DOI:** 10.3390/ijms232213878

**Published:** 2022-11-10

**Authors:** Dimitra C. Bobori, Konstantinos Feidantsis, Anastasia Dimitriadi, Nefeli Datsi, Panagiotis Ripis, Stavros Kalogiannis, Ioannis Sampsonidis, Georgia Kastrinaki, Nina Maria Ainali, Dimitra A. Lambropoulou, George Z. Kyzas, George Koumoundouros, Dimitrios N. Bikiaris, Martha Kaloyianni

**Affiliations:** 1 Laboratory of Ichthyology, Department of Zoology, School of Biology, Aristotle University of Thessaloniki, GR-541 24 Thessaloniki, Greece; 2 Laboratory of Animal Physiology, Department of Zoology, School of Biology, Aristotle University of Thessaloniki, GR-541 24 Thessaloniki, Greece; 3 Biology Department, University of Crete, GR-700 13 Herakleion, Greece; 4 Department of Nutritional Sciences and Dietetics, International Hellenic University, GR-574 00 Thessaloniki, Greece; 5 Laboratory of Inorganic Materials, CERTH/CPERI, GR-570 01 Thessaloniki, Greece; 6 Laboratory of Environmental Pollution Control, Department of Chemistry, Aristotle University of Thessaloniki, GR-541 24 Thessaloniki, Greece; 7 Laboratory of Polymer Chemistry and Technology, Department of Chemistry, Aristotle University of Thessaloniki, GR-541 24 Thessaloniki, Greece; 8 Center for Interdisciplinary Research and Innovation (CIRI-AUTH), Balkan Center, GR-570 01 Thessaloniki, Greece; 9 Department of Chemistry, International Hellenic University, GR-654 04 Kavala, Greece

**Keywords:** FTIR spectra, oxidative stress biomarkers, metabolomics, apoptosis, zebrafish, perch

## Abstract

The massive accumulation of plastics over the decades in the aquatic environment has led to the dispersion of plastic components in aquatic ecosystems, invading the food webs. Plastics fragmented into microplastics can be bioaccumulated by fishes via different exposure routes, causing several adverse effects. In the present study, the dose-dependent cytotoxicity of 8–10 μm polypropylene microplastics (PP-MPs), at concentrations of 1 mg/g (low dose) and 10 mg/g dry food (high dose), was evaluated in the liver and gill tissues of two fish species, the zebrafish (*Danio rerio*) and the freshwater perch (*Perca fluviatilis*). According to our results, the inclusion of PP-MPs in the feed of *D. rerio* and *P. fluviatilis* hampered the cellular function of the gills and hepatic cells by lipid peroxidation, DNA damage, protein ubiquitination, apoptosis, autophagy, and changes in metabolite concentration, providing evidence that the toxicity of PP-MPs is dose dependent. With regard to the individual assays tested in the present study, the biggest impact was observed in DNA damage, which exhibited a maximum increase of 18.34-fold in the liver of *D. rerio*. The sensitivity of the two fish species studied differed, while no clear tissue specificity in both fish species was observed. The metabolome of both tissues was altered in both treatments, while tryptophan and nicotinic acid exhibited the greatest decrease among all metabolites in all treatments in comparison to the control. The battery of biomarkers used in the present study as well as metabolomic changes could be suggested as early-warning signals for the assessment of the aquatic environment quality against MPs. In addition, our results contribute to the elucidation of the mechanism induced by nanomaterials on tissues of aquatic organisms, since comprehending the magnitude of their impact on aquatic ecosystems is of great importance.

## 1. Introduction

The pollution of aquatic environments with plastic waste is constantly increasing. Polymeric materials or plastics derived from fossil fuel-based chemicals find use in almost all aspects of human activities including packaging materials, electronics, construction, transportation, health care, and agriculture. It is estimated that plastics floating on the ocean surface [1,2] will outweigh fishes in the ocean by 2050 [3], since over 1.5–4.1% of the total plastic production flows into the oceans [4]. Moreover, 109 million tons of plastic waste has so far been accumulated in rivers [5], while they are also abundant in several lakes [6].

The transfer of microplastics (MPs) via the food chain is also gaining research interest [7,8]. Laboratory research and field works have documented the ability of organisms of different trophic levels to absorb, concentrate, and bioaccumulate MPs after short-term or chronic exposure, thus posing a threat to human health via the consumption of contaminated food [9].

Microplastic toxicity is related, among others, to the size [10,11,12] and polymer type [13,14], the concentration [15], the duration of exposure [16] as well as the presence of toxic additives [17]. Generally, smaller particle sizes and higher doses appear to induce more acute toxic effects to several organisms [12,18]. Once MPs are ingested by aquatic organisms, they tend to accumulate and have the potential to cause adverse effects such as inhibited growth and development [19,20], reduced feeding activity [21], energy disturbance [8], endocrine disruption [22], immunity, oxidative stress [23], genotoxicity [24], neurotransmission malfunction [8,23], and even mortality [19]. High dose and chronic exposure can lead to higher cellular uptake, causing higher toxicities [25]. Dose-dependent cytotoxicity was also shown [26]. Nevertheless, high MP concentrations do not seem to produce adverse effects in every case [27].

Aside from the toxicity reasons, the MP behavior in the aquatic environment is mainly affected by two critical factors: the MP shape and density. Alterations in these two characteristics may cause the different dispersion of the MPs in several parts of the aquatic environment, thus affecting their availability to organisms of the several trophic levels [28].

Polypropylene (PP) is a synthetic and thermoplastic polymer belonging to the family of the most common used plastics, since it covers a wide spectrum of routine applications [29]. Its well-known character is mainly attributed to PP’s exceptional properties, enclosing chemical inertness, toughness, flexibility, and long-term resistance to a variety of harsh conditions anticipated, evidencing its applicability in both commercial and domestic products such as textiles, stationery, optoelectronics, packaging, toys, automotive parts, medical devices, and laboratory apparatus [30,31]. Hence, it is not surprising that PP represents a 19.7% market-share in the global plastic demand (Plastics–the Facts 2021), rendering PP’s production as the second largest polymer marketplace worldwide [32], while it is anticipated to reach 21.65% by 2050, generating a major source of concern for the academic committee [33,34].

Taking into account that PP is the second most extensively produced and utilized polymer type globally, the continuous fabrication of PP-based products and the irregular disposal processes constitute PP as an emerging environmental pollutant. In light of the above, PP is a dynamic candidate toward MP pollution after ending up in oceans and aquatic ecosystems as expected [35], while its exposure to several environmental stresses (UV exposure, moisture, mechanical abrasion, etc.) leads to its further fragmentation [29,36]. Considering the density of virgin PP, which fluctuates within the range 0.90–0.92 g/cm^3^, it tends to float in seawater and thus freshwater organisms are more likely to consume them when existing in the microscale. Consequently, fish may ingest MPs in a direct or an indirect (detected in the prey) pathway. Despite some studies that have been published reporting propylene gas-related toxicity evaluations [37], the research evaluating the direct toxicity of PP MPs is still limited.

Since data on the effects of different concentrations of MPs are ambiguous, the purpose of the present study was to understand the cellular toxicity that two different doses of PP MPs exert on two freshwater fish species used as model organisms, zebrafish (*Danio rerio*) and perch (*Perca fluviatilis*) [12,38,39]. Biomarkers such as the neutral red retention assay, lipid peroxidation, DNA damage, apoptosis, autophagy, and ubiquitin derivatives as well as metabolite analysis have largely been used for the assessment of environmental health and the estimation of environmental risk [19,40,41,42,43,44].

In this study, the effects of the ingestion of PP-MPs sized 8–10 μm on the liver and gills of adult specimens of *D. rerio* and *P. fluviatilis* were assessed. Thus, two experiments were set up to study the dose-dependence cytotoxicity of PP-MPs, after feeding fish with food enriched with particles at concentrations of 1 mg/g (low dose) and 10 mg/g dry food (high dose). Hence, a battery of biomarkers was used reflecting the oxidative stress, physiology, tissue function, and metabolic profile responses of the studied animals. The outcome of the present study is expected to contribute to the understanding of the biochemical and molecular mechanisms that PP-MPs activate on fish tissues. Furthermore, by studying the oxidative stress parameters after the effect of PP-MPs on fish, we can suggest a battery of biomarkers as early-warning tools against MPs and thus may help to deduce and extrapolate the risk MPs exert to the aquatic environment and furthermore to human health.

## 2. Results and Discussion

### 2.1. Characterization—Internalization of PP-MPs

The FTIR spectra of the controlled and exposed gills and liver samples (Supplementary Figure S1) exhibited similar peaks, which are mainly attributed to DNA and RNA proteins, present both in zebrafish and perch organs. For the ATR-FTIR measurements, the received tissue after dehydration was subjected to analysis; for the zebrafish organ characterization, the whole gills and liver tissue were analyzed, while in the perch, where the organs are larger than zebrafish, a part of the exposed tissue was analyzed. In the case of the presence of PP in the tissue, the concentration of PP was low related to the whole organ placed for analysis in the ATR-FTIR instrument, thus the respective PP peaks were expected to have low intensities, which were overwhelmed by the N–H related bond peaks at the region of 2920 and 2950 cm^−1^. In order to study potential peak variations in such a case, a second derivative analysis was performed [45], where by this method, the potential PP peak contribution to the spectrum could be verified qualitatively; the negative values of the second derivative showed increased intensity in the respective wavenumbers, thus downside peaks were related to the presence of PP.

**Figure 1:**
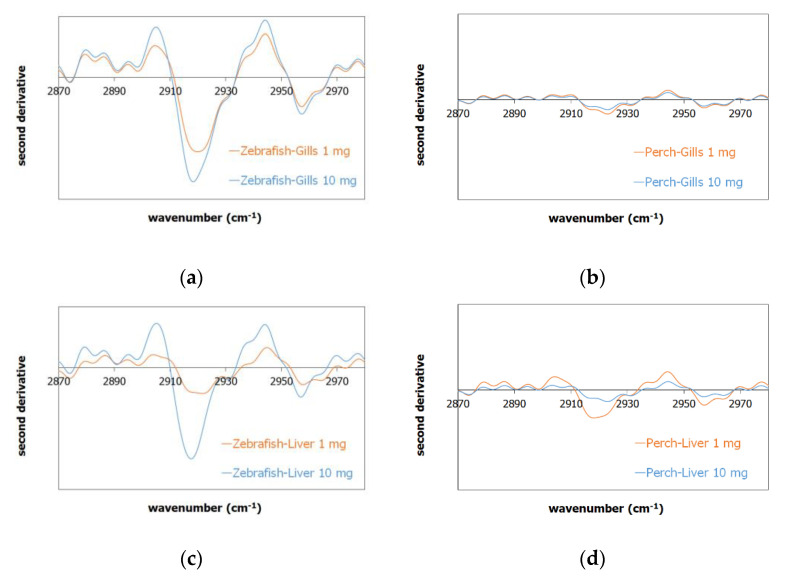
Second derivative of the FTIR spectrum of the exposed sample to 1 mg of PP (orange line) and 10 mg of PP (blue line) in the (**a**) zebrafish gills, (**b**) perch gills, (**c**) zebrafish liver, and (**d**) perch liver, where the downside peaks at 2920 and 2950 cm^−1^ are related to the presence of PP.

The exposed gill samples of zebrafish in Figure 1a are denoted as zebrafish-gills-1 mg (orange line) and zebrafish-gills-10 mg (blue line) exposed to 1 and 10 mg of PP-MPs, respectively; a similar annotation follows the perch (Figure 1b) and liver samples (Figure 1c,d). The comparison of the gills second derivative spectra in Figure 1a and of the liver second derivative in Figure 1c for zebrafish showed an increased concentration of PP-MP for the zebrafish gills and liver exposed to 10 mg of PP-MPs compared to the 1 mg exposed fishes. The respective gills and liver of the perch samples (Figure 1b,d) showed similar intensity at 2920 and 2950 cm^−1^, which can be attributed to a similar concentration of PP in the samples. It should be denoted though, that in the perch case compared to the zebrafish, the respective organs were larger, thus the PP concentration was expected to be lower than the zebrafish measurements.

The internalization of MPs/NPs by the gastrointestinal, respiration airway as well as immune and other miscellaneous cell types has previously been described [46]. Since hemocytes as immune cells act as doorkeepers of the body, they help in the clearance of pathogens or xenobiotics. Therefore, MPs can be taken up by immune cells via phagocytosis or micropinocytosis, while smaller particles can be internalized in the cells employing clathrin/caveolae-mediated endocytosis or clathrin/caveolae independent internalization [47]. According to Karavelidis et al. [48], biomaterial internalization in the immune cells is assisted by enzymes, reactive oxygen species (ROS), and acids secreted by macrophages and other cell types. As a result, polymeric fragments are released into the intercellular matrix that are absorbed by macrophages, giant cells, and indigenous fibroblasts [49,50].

### 2.2. Molecular and Biochemical Responses

#### 2.2.1. NRRT

**Figure 2:**
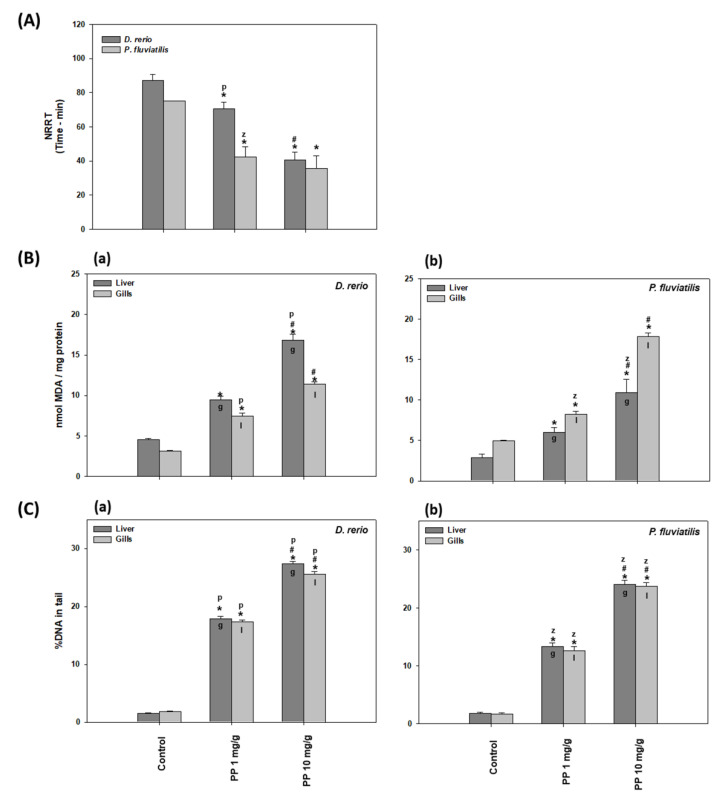
(**A**) NRRΤ levels in the *Danio rerio* and *Perca fluviatilis* control and treated with 1 mg/g and 10 mg PP-MPs/g dry food. (**B**) MDA concentrations (mean ± SD nmol/mg protein) and (**C**) percentage (%) of DNA damage in the tail (mean ± SD) in the liver and gills of the *Danio rerio* (**a**) and *Perca fluviatilis* (**b**) control and dietary exposed to 1 mg PP-MPs /g and 10 mg PP-MPs/g dry food. %DNA in tail and olive moment in positive control data (1 μΜ H_2_O_2_) were 28.3 ± 5.2 and 40 ± 6.3, respectively. Asterisk (*) denotes significant differences (*p* < 0.05) compared to the control group, dash (#) denotes significant differences (*p* < 0.05) between the two examined concentrations: 1 mg PP-MPs/g and 10 mg PP-MPs/g, lower case letters “z” and “p” denote significant differences (*p* < 0.05) between the two examined species *D. rerio* and *P. fluviatilis,* respectively, while lower case letters “g” and “l” denote within species significant differences (*p* < 0.05) between the gills and liver, respectively.

The NRRΤ levels in the *D. rerio* and *P. fluviatilis* hemocytes under the effect of both PP-MP concentrations are shown in Figure 2A. All exposed cells exhibited lower NRRT values than the controls. In particular, the ΝRRT for the control zebrafish was 87 min, which was lowered to 70 and to only 40 min for the exposed fish to 1 and 10 mg/g PP-MPs, respectively. In parallel, the NRRT for *P. fluviatilis* was 75 min for the control fish and in the presence of 1 and 10 mg/g of PP-MPs, this time value decreased to 42.5 and 35.6 min, respectively. Thus when both fish were exposed to 1 mg/g of PP-MPs, the perch was more susceptible than zebrafish, where a 43.3% decrease in the NRRT value was observed compared to the 18.72% decrease observed for zebrafish. The 10 mg/g concentration showed an almost equal decrease in both fish species (57.86 % and 52.5% decrease in the zebrafish and in the perch, respectively) compared to the control. Hence, this indicates that in *D. rerio* hemocytes, lysosomal membrane destabilization is dose-dependent. In *P. fluviatilis*, at both concentrations tested, a statistically similar NRRT reduction was shown, which was also similar to the reduction observed in *D. rerio* at the high dose, indicating that probably in *P. fluviatilis*, the maximal NRRT reduction had already been achieved at the low dose.

Lysosomal membrane stability is related to general stress as well as to toxicological and pathological responses in organisms. Lysosomal overload with xenobiotics could cause membrane damage, leading to their destabilization, an increase in autophagy, the release of hydrolases in the cytoplasm, and finally, necrosis. NRRT is considered as a sensitive biomarker for the early warning of pollution in ecosystems [38,51]. More specifically, NRRT has been used as a marker of lysosomal destabilization after MP exposure in freshwater fish [41,42,43]. Our results, with regard to the dose response of lysosomal membrane stability against MP concentration, are in line with those of Canesi et al. [52], where mussels of *Μ. galloprovincialis* were exposed to three PS microplastic concentrations of 1, 5, 50 μg/mL of water. They observed a greater lysosomal destabilization to those animals exposed to the higher PS concentration. In addition, the exposure of oysters of *Crassostrea gigas* to 6 μm of PS microplastics of three different concentrations for 80 days revealed that oysters exposed to the higher concentration exhibited increased lysosomal destabilization [53].

#### 2.2.2. Lipid Peroxidation

In the present study, after the exposure of fish for 21 days to 1 and 10 mg/g of 8–10 μm of PP-MPs, the MDA levels were significantly increased in the gills and liver cells of both fish in comparison to the controls (Figure 2B). In this case, the harmful effects induced more lipid peroxidation at the high dose, indicating dose dependency. In particular, in all tissues, the MDA values at 1 mg/g PP-MPs increased by a fold in the range of 1.66–2.37 and at 10 mg/g PP-MPs by 3.60–3.78-fold compared to the controls.

MPs have been shown to induce the production of free radicals in animal tissues [23,54]. Lipid peroxidation can be described as a procedure where oxidative parameters as free radicals (ROS) attack membrane lipids with double bonds, especially polyunsaturated fatty acids (PUFAs), leading to the production of aldehydes and may contribute to the disruption of membrane integrity [55]. The terminal product of lipid peroxidation, malondialdehyde (MDA), is formed by the breakdown of polyunsaturated fatty acids. MDA is used as an indicator of oxidative stress in a broad number of aquatic and terrestrial animals [38,40,42,56,57,58,59,60,61]. Our results showed that PP-MPs induced oxidative stress to fish tissues, where a larger concentration was more potent in inducing lipid peroxidation than a smaller one. This concentration dependance of MPs has also been reported by Barboza et al. [8], who exposed *Dicentrarchus labrax* to 0.26 mg/L and 0.69 mg/L fluorescent red polymer microspheres, 1–5 μm in diameter, for 96 h. They observed that the oxidative damage was evident in the larger but not in the smaller concentration of MPs tested. Μoreover, it is remarkable that the MDA levels estimated in the present work seemed to be similar or higher than the corresponding levels following exposure to other toxic substances such as the pesticide chlorpyrifos on mussels [62].

#### 2.2.3. DNA Damage

DNA damage in the liver and gills of *D. rerio* and *P. fluviatilis* under the effect of PP-MPs is shown in Figure 2C. Our results revealed that both concentrations of PP-MPs caused significant oxidative injury to the DNA in both the gills and liver cells. The greater the concentration, the greater the damage they induced to both tissues studied.

Regarding the tissue sensitivity to MPs in the zebrafish, the increase in DNA damage was greater in the liver compared to the gills (12.02-fold increase in the liver versus 9.64-fold increase in gills at 1 mg/g, and 18.34-fold increase in the liver versus a 14.23 increase in the gills at 10 mg/g of MPs) (Figure 2Ca). Moreover, both concentrations resulted in significant inter-tissue differences in perch (Figure 2Cb).

Among the biological molecules, DNA comprises a target of free radicals and results in gene mutations, chromosomal alterations, and micronucleus formation, a state that is considered as genotoxic. The genotoxic effects caused by pollutants are attributed to the overproduction of ROS [63]. Apart from ROS, which are responsible for the increased DNA damage after MP exposure [64], genotoxicity due to MP exposure has also been related to the inhibition of repair enzymes [65]. DNA damage thus constitutes an important biomarker in ecotoxicological studies [40,42,43,58,62,66,67,68]. In agreement with our results, recently, many investigators have reported a dose-dependency response in genotoxicity due to MP exposure [68].

#### 2.2.4. Ubiquitination

**Figure 3:**
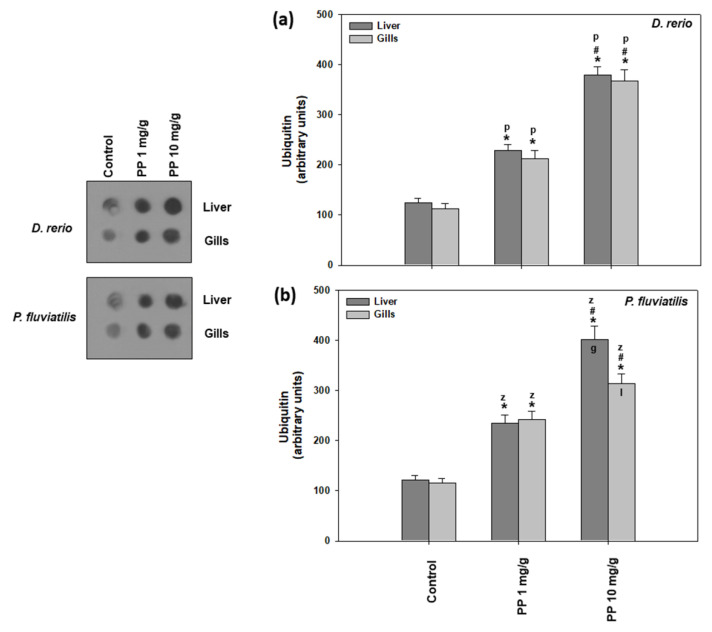
Ubiquitin conjugates (mean ± SD) in the liver and gills of *Danio rerio* (**a**) and *Perca fluviatilis* (**b**), control and dietary exposed to 1 mg PP-MPs /g and 10 mg PP-MPs/g dry food. Asterisk (*) denotes significant differences (*p* < 0.05) compared to the control group, dash (#) denotes significant differences (*p* < 0.05) between the two examined concentrations: 1 mg PP-MPs/g and 10 mg PP-MPs/g, lower case letters “z” and “p” denote significant differences (*p* < 0.05) between the two examined species *D. rerio* and *P. fluviatilis,* respectively, while lower case letters “g” and “l” denote within species significant differences (*p* < 0.05) between the gills and liver, respectively.

Our results on both the fish response to PP-MPs with regard to ubiquitin are presented in Figure 3 In all cases, the higher PP-MP concentration caused a higher increase in ubiquitination in comparison to the controls, which suggests that a higher PP-MP concentration is more potent than the lower one in affecting ubiquitination. Specifically, in animals exposed to 10 mg/g of PP-MPs, an approximately 3-fold increase in ubiquitination was observed at the 21st day in relation to the controls and an approximately 2-fold increase in comparison to the smaller concentration used. In zebrafish, no tissue specificity was observed (Figure 3a). However, in the case of perch, the liver seemed to respond with more sensitivity than the gills, but only at the higher concentration (Figure 3b).

Ubiquitination is widely used in the investigation of cellular stress caused by environmental pollutants [38,40,42,43,58,69]. Specifically, polystyrene microplastics cause elevated levels of ubiquitin levels in several tissues of fresh water fish species such as *D. rerio* and *P. fluvitilis* [38,43]. Other pollutants such as nanoparticles have also exerted significant effects in the ubiquitin system of both terrestrial and aquatic animals. Iron oxide NPs caused increased ubiquitin conjugate levels in the Mediterranean mussel *Mytilus galloprovincialis* [40]. The same effect has also been exhibited in the land snail *Cornu aspersum* when exposed to CuO, ZnO, and TiO_2_ nanoparticles [42,67] and in *D. rerio* and *Carassius gibelio* that ingested TiO_2_ nanoparticles in their food [42]. Our present results, together with the existing literature, show that in the presence of pollutants such as microplastics as well as nanoparticles, selected cellular proteins are tagged by ubiquitin, in order for degradation by ATP-dependent 26S proteasomes [70]. The ubiquitin pathway is also involved in significant cellular functions such as apoptotic cell death and antioxidant defense [71].

#### 2.2.5. Autophagy

**Figure 4:**
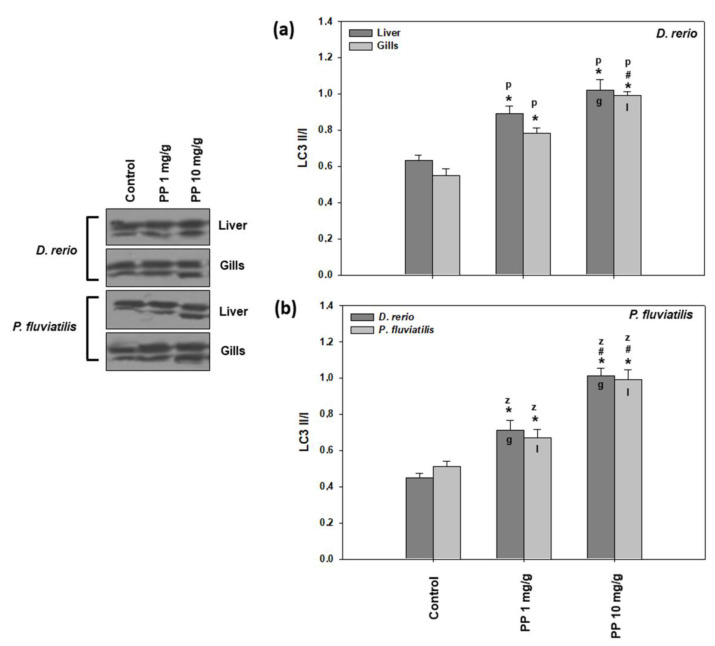
LC3 II/I levels (mean ± SD) in the liver and gills of the *Danio rerio* (**a**) and *Perca fluviatilis* (**b**) control and dietary exposed to 1 mg PP-MPs/g and 10 mg PP-MPs/g dry food. Asterisk (*) denotes significant differences (*p* < 0.05) compared to the control group, dash (#) denotes significant differences (*p* < 0.05) between the two examined concentrations: 1 mg PP-MPs/g and 10 mg PP-MPs/g, lower case letters “z” and “p” denote significant differences (*p* < 0.05) between the two examined species *D. rerio* and *P. fluviatilis*, respectively, while lower case letters “g” and “l” denote within species significant differences (*p* < 0.05) between the gills and liver, respectively.

As can be seen in Figure 4, the highest increase in LC3II/I was measured in both tissues of both fish exposed to 10 mg/g of PP-MPs (in the range of 1.62–2.24-fold increase) in comparison to the lower concentration of PP-MPs (in the range of 1.31–1.58-fold increase).

In parallel to lipid peroxidation and DNA damage, autophagy is also associated with oxidative stress. Autophagy has also been suggested as a sensitive biomarker for the detection of land pollution [66,72]. In agreement with our results, increased levels of LC3II/I due to magnetite NPs, PE-MPs, and PS-MP exposure of *P. fluviatilis* was also observed [8,12,38,57].

**Figure 5:**
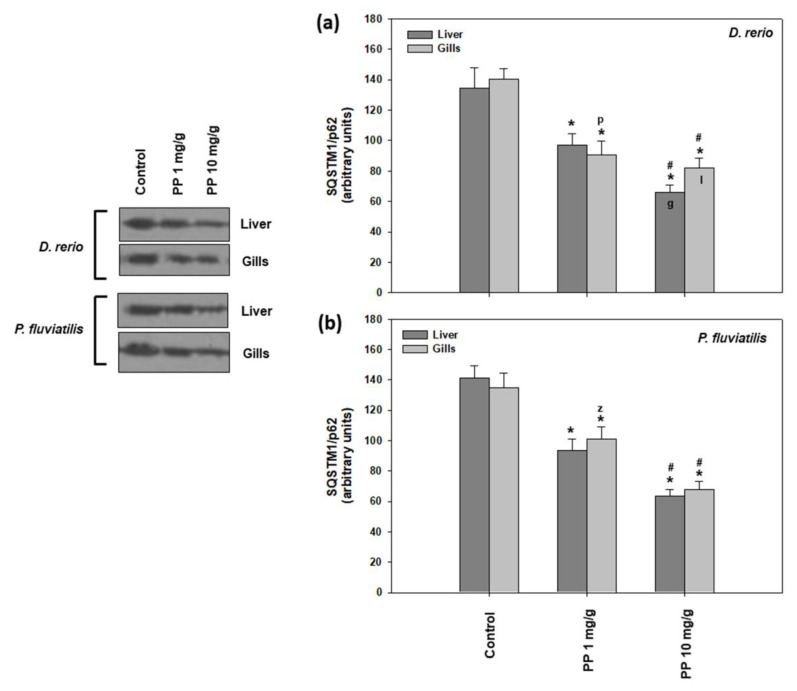
SQSTM1/p62 levels (mean ± SD) in the liver and gills of the *Danio rerio* (**a**) and *Perca fluviatilis* (**b**) control and dietary exposed to 1 mg PP-MPs/g and 10 mg PP-MPs/g dry food. Asterisk (*) denotes significant differences (*p* < 0.05) compared to the control group, dash (#) denotes significant differences (*p* < 0.05) between the two examined concentrations: 1 mg PP-MPs /g and 10 mg PP-MPs/g, lower case letters “z” and “p” denote significant differences (*p* < 0.05) between the two examined species *D. rerio* and *P. fluviatilis*, respectively, while lower case letters “g” and “l” denote within species significant differences (*p* < 0.05) between the gills and liver, respectively.

Regarding the other autophagy marker studied, SQSTM1/p62, the results showed a similar pattern (Figure 5), as in the case of LC3 II/I, where the higher concentration of PP-MPs caused a more potent response to both tissues of both fishes than the lower one. Specifically, in zebrafish, the 1 mg/g concentration caused a 28.1% and 35.1% decrease, while in the 10 mg/g concentration, there was a 51.1%- and 41.5%-fold decrease in the liver and gills, respectively (Figure 5a). Concerning perch, the 1 mg/g concentration caused a 33.8% and 24.9% decrease, while the 10 mg/g concentration a 55.1% and 49.7% decrease in the liver and gills, respectively (Figure 5b). PP-MPs triggered the autophagic machinery in both tissues of the zebrafish and perch. Hence, our results strongly indicate dose-dependent increased autophagy in both tissues of the PP-MP exposed fish. Generally, differences in tissue and interspecies responses were not substantial, except for the highest concentration in zebrafish, with the liver being more sensitive than gills (Figure 5a).

Autophagy contributes to sustaining cellular homoeostasis, in order to enable adaptation to hostile circumstances. It regulates cell survival, differentiation, and growth as well as cellular defense and may serve as either protective or death-promoting purposes. LC3 (autophagy microtubule-associated protein 1 light chain 3) is an indicator of autophagosome formation and is therefore upregulated in the process of autophagy in diseased cells [73]. On the other hand, sequestrosome 1 (SQSTM1/p62) is a multifunctional protein linked to signal transduction, protein degradation, and cell transformation that is downregulated in the process of autophagy [73]. Similar to the results presented herein, MPs have been shown to trigger the autophagic process in several animal models [38,42,43,74,75].

#### 2.2.6. Apoptosis

**Figure 6:**
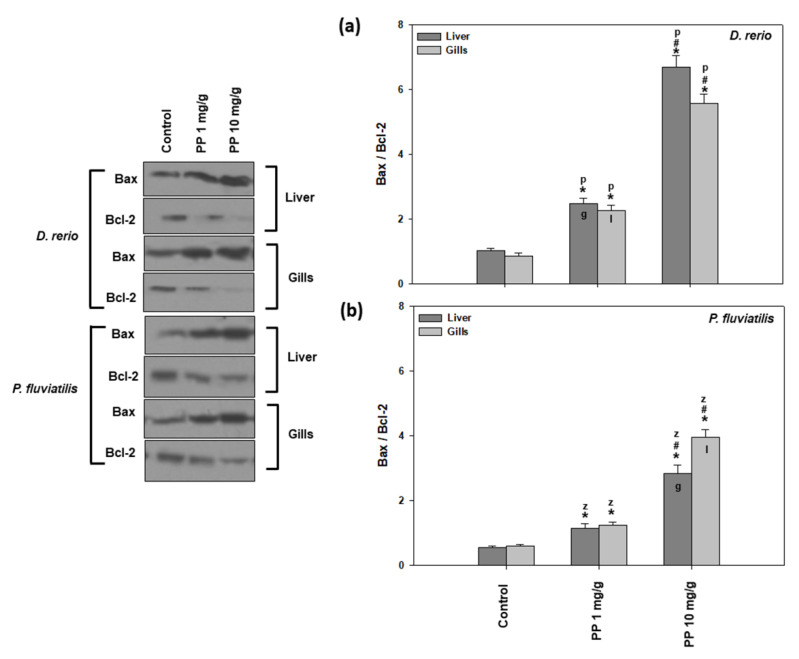
Bax/Bcl-2 ratio (mean ± SD) in the liver and gills of the *Danio rerio* (**a**) and *Perca fluviatilis* (**b**) control and dietary exposed to 1 mg PP-MPs/g and 10 mg PP-MPs/g dry food. Asterisk (*) denotes significant differences (*p* < 0.05) compared to the control group, dash (#) denotes significant differences (*p* < 0.05) between the two examined concentrations: 1 mg PP-MPs/g and 10 mg PP-MPs/g, lower case letters “z” and “p” denote significant differences (*p* < 0.05) between the two examined species *D. rerio* and *P. fluviatilis*, respectively, while lower case letters “g” and “l” denote within species significant differences (*p* < 0.05) between the gills and liver, respectively.

**Figure 7:**
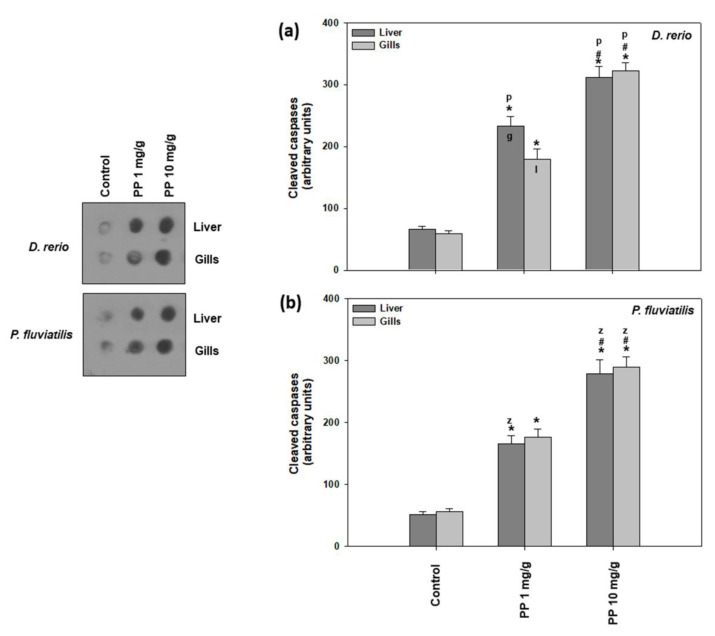
Cleaved caspase levels (mean ± SD) in the liver and gills of the *Danio rerio* (**a**) and *Perca fluviatilis* (**b**) control and dietary exposed to 1 mg PP-MPs /g and 10 mg PP-MPs /g dry food. Asterisk (*) denotes significant differences (*p* < 0.05) compared to the control group, dash (#) denotes significant differences (*p* < 0.05) between the two examined concentrations: 1 mg PP-MPs /g and 10 mg PP-MPs/g, lower case letters “z” and “p” denote significant differences (*p* < 0.05) between the two examined species *D. rerio* and *P. fluviatilis*, respectively, while lower case letters “g” and “l” denote within species significant differences (*p* < 0.05) between the gills and liver, respectively.

In the present study, two apoptosis indicators were measured, the ratio of Bax/Bcl-2 (Figure 6) and the cleaved caspase activation (Figure 7). Both PP-MP concentrations significantly increased apoptotic indicators in the liver and gills of zebrafish and perch compared to the control.

As observed in the other parameters/biomarkers measured, the 10 mg/g concentration caused a statistically significant higher increase in the ratio of Bax/Bcl-2 in relation to the lower concentration of 1 mg/g. Regarding the tissue differences, the *P. fluviatilis* gills (6.68-fold increase) were found to be more vulnerable compared to the liver (5.07-fold increase) when the fish were fed with the 10 mg/g of PP-MPs (Figure 6b). However, the opposite pattern was observed for the zebrafish tissues’ response at the lower concentration studied (Figure 6a).

As for the activation of cleaved caspases (Figure 7), the other marker of apoptosis studied, once again, both fish tissues were more intensively affected by the higher PP-MP concentration compared to the lower one. Specifically, in zebrafish, the high dose caused a 4.73- and 5.47-fold increase in the liver and gills, respectively, compared to the lower concentration, which resulted in a 3.04- to 3.54-fold increase (Figure 7a). In perch, 10 mg/g provoked a 5.36- and 5.09-fold increase in the liver and gills, respectively, while the 1 mg/g concentration showed a 3.18- and 3.12-fold increase (Figure 7b). Our results show that the apoptotic cascade is triggered as both the Bax/Bcl-2 ratio and caspases levels are activated after exposure to PP-MPs, in a dose dependent profile. Although in general, significant interspecies differences were observed in both tissues and concentrations, inter-tissue differences were significant only for zebrafish exposed to the low dose (Figure 7).

In agreement with our results, PP-MPs induced dose-and time-dependent ROS mediated apoptotic responses in zebrafish [76,77], suggesting that oxidative stress also causes damage to the organelles such as lysosomes, nucleus, and mitochondria, causing additional enhancement in the stress signal, probably via caspase activation, which leads to DNA fragmentation and apoptosis. Specifically, apoptosis is activated through a signaling pathway that involves the Bax and Bcl-2 as well as increased levels of caspase. The increased ratio of Bax/Bcl2 observed in our study was probably due to diminished Bcl-2, as a result of the inactivation of the anti-apoptotic proteins of the Bcl- 2 family [78]. Our results revealed that the same signaling pathway was activated after both MP concentrations tested, since the responses of the apoptotic indicators increased in a dose response behavior together with increased MP concentration. In accordance, in the PC12 cells, the highest concentration of pollutants impacted on the highest levels of ROS, and could have a faster influence on the mitochondria [79]. On the other hand, according to Châtel et al. [80], in mussel, the concentration of pollutants could influence whether the apoptotic pathways were dependent or not dependent on caspase 3. In addition, in accordance with our results, the exposure of freshwater fish species such as *D. rerio*, *P. fluviatilis*, and *C. carpio* to pollutants such as TiO_2_ NPs, magnetite NPs, PS-MPs, and PE MPs [12,38,42,57] exhibited increased levels of apoptotic activity in all of the examined tissues.

### 2.3. Metabolomics

The levels of most metabolites in the gills and liver of the exposed *P. fluviatilis* exhibited a considerable decrease, as shown in Table 1. Two metabolites that were greatly affected were tryptophan and nicotinic acid, whose levels decreased by 52 to 96%, mostly in the gill tissue but also in the liver. Interestingly, tryptophan is an essential amino acid in fish [81] and the parent compound for the biosynthesis of nicotinic acid and nicotinamide in the liver (smpdb.com). Both nicotinic acid and nicotinamide are also important contributors in maintaining the health of the central nervous system [81], while nicotinamide is a component of the widely used coenzymes NAD and NADP, both central in energy metabolism. Additionally, a considerable decrease was observed in the levels of the neurotransmitter γ-aminobutyric acid and the other aromatic amino acids phenylalanine and tyrosine. Tryptophan is also the metabolic precursor of the neurotransmitter serotonin and the hormone melatonin, whereas phenylalanine and tyrosine are precursors of the catecholaminic neurotransmitters tyramine, dopamine, and norepinephrine, which act as adrenalin-like substances, and of epinephrine itself (smpdb.com). Regarding nucleic acid metabolism, apparently associated with DNA damage, which was increased in the samples of all treatments, the metabolites ribose, adenine, adenosine, guanine, and cytosine exhibited remarkable reductions in the liver, mostly in fish exposed to the high dose. In the gills, uracil and adenosine almost tripled at both doses. Similarly, metabolites related to energy metabolism, glucose/galactose, fumaric acid, ribose, carnitine, and nicotinamide also exhibited a considerable decrease, mainly in the liver. This could be attributed to the function of each tissue; the liver is crucial in distributing energy metabolites within the body, hence their reduction, due to the observed mitochondrial dysfunction, is reflected strongly in the liver metabolome. The above observations indicate possible alterations in energy metabolism and nervous function, in accordance with Dimitriadi et al. [43], who reported reduced swimming performance in fish exposed to polystyrene MPs.

Although the gills are vital for the oxygenation of the whole fish and their regular function is necessary for maintaining life, considerable alterations of the metabolome were also observed in the gills. Regarding amino acid metabolism in the gills, at a low dose, the levels of all amino acids, except glutamic acid, decreased in relation to the control, whereas at a high dose, the levels of tyrosine increased almost 4-fold, phenylalanine and leucine/isoleucine almost 3-fold, proline, valine, and alanine almost doubled, and citrulline, another metabolite related to the urea cycle and nitrogen metabolism, increased almost 2.5-fold. Similar increases in the liver were observed only in glutamic acid, which doubled, and in tyrosine, which increased impressively almost 5-fold at a low dose. Although little is known for the interpretation of these observations, the increase in many amino acids and citrulline, as in the gills at high dose, could be related to the observed increased ubiquitination and autophagy, which trigger protein breakdown into their constitutive amino acids. The fact that increased levels of these metabolites are more apparent in the gills could be attributed to the high metabolic rate of the liver and its role as a major distributor to other organs.

Comparing the effects of the two levels of exposure in the gills, it was observed that only 14 metabolites exhibited a change greater than 25% at the low dose compared to 22 at the high dose, probably indicating an increased effect. In the liver, the metabolites exhibiting a change greater than 25% at the low and high dose were 25 and 20, respectively. It is noteworthy that at the high dose, the levels of all 20 changed metabolites were decreased compared to the control, which could be indicative of a substantial reduction in the metabolic rate in the liver. However, conclusions from this observation should be drawn carefully, since although changes in the levels of metabolites can be valuable in identifying early metabolic responses, the part of metabolism affected, and a physiology deviating from normal, a direct quantitative correlation to the severity of tissue dysfunction would require a detailed knowledge of each metabolite’s function and role in the organ’s metabolism.

Recently, zebrafish has been used in combination with metabolomics to represent aquatic vertebrates in a number of toxicological studies [82,83,84], but also to study metabolome changes in several adverse conditions such as acute organophosphorus poisoning [85], diabetes mellitus [86], and drug-induced renal tubular injury [87]. Regarding MPs, Qiao et al. recently demonstrated the adverse effects of MP ingestion on the gut metabolome of zebrafish, revealing that MPs induced metabolic disorders on amino acid and lipid metabolism [88]. Similar studies have been performed on the adverse effects of MPs on the microbiome and metabolism of zebrafish and on their heart tissue [89], the effects of polyethylene MP size on the gills and liver of perch [12], and the possible toxicity enhancements that MPs may induce on chemical species such as synthetic phenolic antioxidants [84]. Moreover, since MPs have been detected in several wild freshwater fish populations [90,91], the use of such organisms for toxicological studies [12,38,92] has gained research attention, bearing in mind the important role they have as a major protein source in the human diet.

## 3. Materials and Methods

### 3.1. Preparation of PP-MPs

For the preparation of PP-MPs, a 1% *w*/*v* PP (ECOLEN^®^ HZ40 P, Hellenic Petroleum, Marousi, Greece) solution was prepared in a mixture of xylene/toluene (50/50 *v*/*v*) after 1 h of reflux. An aqueous 1% *w/v* poly (vinyl alcohol) (PVA) (87–89%, CAS No. 9002–89-5, Mw = 13,000–23,000, Aldrich^®^, Burlington, MA, USA) solution was also prepared, after 1 h of magnetic stirring, at 80 °C. Afterward, the polymer solution was divided in 5 mL of volume parts and added with a Pasteur pipette in well-separated drops into the PVA aqueous solution (50 mL of volume intervals), while intensive mechanical stirring took place for 2 min (Ultra-Turrax^®^, T10 basic, IKA^®^, Staufen, Germany). The emulsion produced was left to stir magnetically for 24 h at room conditions to ensure the removal of the organic solvent after evaporation. Then, the PP-MPs were finally dispersed into the aqueous environment and collected by double centrifugation (Unicen 21, CE 126, Orto Alresa, Spain) at 4500 rpm for 20 min. The white-like precipitation of the PP-MPs was washed and collected with deionized H_2_O, while drying at 80 °C was followed for 24 h under vacuum. From the SEM micrographs (Figure S2), it was found that the prepared PP microparticles were spherical in shape with particle sizes diameters ranging from 3 to 7 μm.

### 3.2. Animal Maintenance

Adult specimens of *D. rerio* (ZF WT 2 F10, Wageningen Agricultural University, Wageningen, The Netherlands), 6 months old, both sexes, of similar body length (total length: 35.8 ± 6.7 mm, mean ± SD) and weight (wet weight: 0.52 ± 0.24 g, mean ± SD) were obtained from the Department of Biology of the University of Crete. All individuals were acclimatized in aerated fish tanks of 30 L (1 individual per 3 L) with filtered water pump circulation, under a 14:10 light:dark cycle, at 26 ± 1 °C temperature, 7.9 ± 0.06 pH, 10.13 ± 0.23 mg/L dissolved oxygen, 453.25 ± 68.85 μS/cm conductivity, and 0.16 ± 0.04 psu salinity.

Adult specimens of *P. fluviatilis* with a similar body length (total length: 12.5 ± 1.18 cm, mean ± SD) and weight (wet weight: 17.7 ± 6.7 g, mean ± SD) were provided from Lake Volvi (Northern Greece) by a commercial fisherman. Perch specimens were immediately placed in 150 L aquariums (six individuals per aquarium) with filtered water pump circulation under a 14:10 light:dark cycle, at 19.9 ± 1.03 °C, pH 8.8 ± 1.01, 9.72 ± 0.67 mg/L dissolved oxygen concentration, 665.87 ± 47.68 μS/cm conductivity, and 0.3 ± 0.02 psu salinity.

Zebrafish were acclimatized for 7 days and fed once per day with commercial flakes (Cichlid Omni Flakes, Ocean Nutrition Europe, Essen, Belgium) while fish excrement in the aquariums was removed manually every day with a net. Perch was acclimatized for 25 days, and their feeding was based on commercial dry *Gammarus pulex* shrimps (Tetra Gammarus, Spectrum Brands Pet, LLC, Blacksburg, VA, USA) applied once per day. The acclimatization time and conditions of the two fish species were different due to differences in their origin and life history characteristics.

### 3.3. Food Preparation and Fish Feeding

We followed the methodology described in detail in Bobori et al. [42] to prepare food enriched with PP-MPs. In brief, the proper quantity of PP-MP particles (8–10 μm in size) were added in commercial food (Cichlid Omni Flakes, Ocean Nutrition Europe, Essen, Belgium for zebrafish and dry shrimps *Gammarus pulex*, Tropical company, for perch) with vortexing and thereafter sonication to obtain the required concentrations of 1 mg and 10 mg PP-MPs/g of dry food. The mixture was then air-dried for 2 h at 50 °C. The flakes were ground again in the mixer to obtain a final powder form. Food for the control groups was prepared following the same procedure but omitting the addition of MPs. Both fish species were fed once per day with food corresponding to 3% of the wet body weight [20,38,93] and inspected during feeding to ensure that all food was consumed.

### 3.4. Experimental Design

Exposed individuals of zebrafish (two groups of 10 individuals per aquarium) were fed with food containing 1 mg and 10 mg PP-MPs/g of dry food, respectively, for 21 days. Control animals (10 individuals) were fed with food with no PP-MP addition. Accordingly, perch specimens (two groups of 6 individuals per aquarium) were fed with food containing the same concentrations of PP-MPs for 21 days, except the control group (six individuals), which was fed with food without the addition of PP-MPs. All in vivo experiments were run in parallel in triplicate to ensure the reproducibility of the results, resulting in a final number of 30 individuals for zebrafish and 18 treatment individuals for perch for each of the PP-MP concentrations selected. During the experimental period, the water quantity and conditions in aquariums were kept constant, while no fish mortality was observed, either in the control or the treated groups for both species.

### 3.5. Tissue Sampling

After the experimental period, zebrafish (control and treated individuals) were anaesthetized in cold water while perch (control and treated individuals) in ethanol and clove oil diluted in water. Thereafter, and always on ice, the gills and liver tissues were extracted from both fish species, placed in Eppendorf tubes, and stored at −80 °C until further biochemical and molecular analyses. Moreover, blood was withdrawn from the heart with a sterile syringe (18G1/2′ needle) and pooled in a tube placed in ice. Then, the blood samples were centrifuged in 3000 rpm for 10 min in 4 °C for hemocytes to be obtained. Subsequently, PS solution (20 mM Hepes, 436 mM NaCl, 10 mM KCl, 53 mM MgSO_4_, 10 mM CaCl_2_, pH 7.3) was added and the hemocyte suspension was immediately analyzed for the neutral red retention assay evaluation.

### 3.6. MPs Characterization

The liver and gill samples from the control and exposed zebrafish and perch individuals were characterized by attenuated total reflectance Fourier transform infrared (ATR-FTIR) spectroscopy. ATR-FTIR analysis is a convenient analysis tool for studying samples directly “as received” in their initial solid state condition without further treatment (e.g., forming a KBr pellet); the received tissues (approximately 2 mg of each liver and gill sample) were dehydrated in ambient conditions for 48 h in a desiccator with silica gel before the analysis and the dehydrated sample was placed in the ATR diamond holder of a Jasco spectrometer (Jasco FTIR-6700, Tokyo, Japan). The spectra were collected in the range of 4000–400 cm^−1^ by 160 scans with 4 cm^−1^ resolution. The second derivative spectrum was calculated by Spectra Manager 2.15.12 software (Jasco Corporation, Tokyo, Japan) based on the process described in Laidou et al. [45].

### 3.7. Molecular and Biochemical Biomarker Assays

The neutral red retention assay (NRRT) was performed according to the method described by Lowe and Pipe [94], after the modification by Dailianis et al. [95]. The time value where at least 50% of fish granular hemocytes present loss of the intracellularly accumulated dye from the lysosomes to the cytosol represents the NRRT. The evaluation of malondialdehyde (MDA), DNA damage, ubiquitin conjugates, autophagic, and apoptotic processes were conducted as described in Kaloyianni et al. [38]. In brief, the lipid peroxidation levels were quantified through thiobarbituric acid reactive substance (TBARS) estimation according to the method described by Niehaus and Samuelsson [96]. DNA damage is expressed as the percentage of DNA in tail. The experimental protocol was carried out according to Singh et al. [97] as modified by Dailianis et al. [95]. Levels of ubiquitination were estimated according to the method described by Hofmann and Somero [98]. Autophagic and apoptotic indicators in both fish species tissues were estimated according to well-established SDS-PAGE/immunoblot protocols that are described in detail in Kaloyianni et al. [38,41].

### 3.8. Metabolomics Analysis

The effects of MP exposure on metabolic processes (such as sugars and lipid metabolism) were assessed by monitoring the levels of several metabolites and performing sample comparisons by means of relative quantification.

#### 3.8.1. Sample Preparation

The extraction of polar metabolites was performed as described in Bobori et al. [12]. In short, 25 mg of tissue was extracted by adding 750 μL of an ice cold mixture of 1:1 methanol:water in a chilled pestle and ground using a chilled mortar. The liquid phase was transferred into an Eppendorf tube and sonicated in an ice bath in short bursts (30 s) with 2 min breaks, for a total of 3 min. Extracts were centrifuged, then supernatant was transferred to a new container and stored at −20 °C until analysis. Before analysis, samples were thawed and L-alanine-3,3,3-d3 was added to a concentration of 5 mg/L. Only perch samples were treated as above; the zebrafish sample size was too small for this procedure.

#### 3.8.2. LC-MS/MS Analysis

Methanol, acetonitrile, NH_4_OH, and CH_3_COONH_4_ were provided from VWR. Metabolite transitions and retention times were verified through several standards purchased from the following companies: Sigma-Aldrich, Alfa Aesar and Acros Organics. Care was taken so that the reagents used were of analytical grade or better.

The analysis of perch fish extracts was performed on a Thermo Scientific™ TSQ Quantum™ Access MAX Triple Quadrupole Mass Spectrometer coupled to an Accela™ 1250 UHPLC pump and an Accela™ autosampler using a Waters™ ACQUITY UPLC BEH Amide Column (1.7 µm, 2.1 × 150 mm).

Mobile phase conditions: solvent A: H_2_O, 0.1% *w/v* NH_4_OH; solvent B: MeCN:MeOH 9:1, 0.1% *w*/*v* NH_4_OH; solvent C: H_2_O 100 mM CH_3_COONH_4_, 0.1% *w*/*v* NH_4_OH. Elution program: start 7.5%A, 87.5%B, 5%C, hold for 4 min, then to 20%A, 75%B, 5%C, over 11 min, then to 55%A, 40%B, 5%C, over 10 min, hold for 2 min (equilibration time: 15 min (total time: 42 min). Column flow: 200 μL/min; column temperature: 60 °C. Injection volume: 4 μL (partial injection); tray temperature: 15 °C. Wash solvent: MeCN:MeOH:H_2_O 2:1:1.

The electrospray source conditions were as follows: capillary temperature 320 °C, vaporizer temperature 280 °C, sheath gas pressure 45 arb, auxiliary gas pressure 10 arb, positive polarity 3500 V, negative polarity 1500 V. Transitions were detected on the mass spectrometer utilizing SRM mode (MS/MS) with compound specific transitions and collision energies while keeping the collision gas pressure stable at 1.5 mTorr.

Performance of the analytical method was assessed as in Bobori et al. [12] by monitoring both the extraction performance and LC-MS/MS analysis precision using six independent extracts of the same tissue run in triplicate and measuring the relative standard deviation (RSD%) for both the extraction and analysis precision. A quality control sample consisting of a mix of the six extracts was run every six injections. Retention times were checked using a global pure standard mix and blanks were used to assess any carry-over effects.

#### 3.8.3. Data Interpretation

In total, data from 32 analytes were used for relative quantitation. The following isomers leucine-isoleucine and glucose-galactose were not separated, thus the sum of the corresponding peak areas was used for the analysis. Data analysis was performed using Thermo Scientific^TM^ LCQUANTM 2.9 QF1. The fold changes of the analytes were calculated, and relative quantitation between the exposed and control perch was performed using peak areas.

### 3.9. Statistical Analysis

The molecular and biochemical analyses results were expressed as mean (±standard deviation, SD) of the mean. Each dataset was tested for normality distribution and equality of variance by the Shapiro–Wilk and Levene’s tests, respectively. The one-way analysis of variance (ANOVA) was used to assess significant differences (*p* < 0.05) between the control and treated specimens per species and between the gills and liver tissues within and between species for both the PP-MP concentrations tested. For biomarkers expressed in arbitrary units, we used the ratios (i.e., the value of a biomarker observed in each tissue of each species divided by the value of the same biomarker in the same tissue and the same species), thus permitting further comparisons between the tissues and species. All statistical analyses were performed using GraphPad Prism 5 and GraphPad Instat 3.0 software.

### 3.10. Ethical Statement

All of the experimental procedures involving the handling and exposure of fish were performed in accordance with Greek (PD 56/2013) and EU (Directive 63/2010) legislation for animal experimentation and welfare. All protocols were approved by the Animal Care Committee of the Biology Department of the University of Crete (Permit Number: 285586, 7 December 2020).

## 4. Conclusions

According to our results, the inclusion of PP-MPs in the feed of *D. rerio* and *P. fluviatilis* hampered the cellular function of the gills and hepatic cells, as observed by the lipid peroxidation, DNA damage, protein ubiquitination, apoptosis, autophagy, and metabolite concentration. Exposure to the higher concentration of PP-MPs consistently affected further activation of all molecular, cellular, and biochemical events studied in the tissues of both fish species, providing evidence that PP-MP toxicity is dose dependent.

Oxidative stress has been widely referred to be linked to MP-induced toxicity. Usually, the cause of oxidative stress is ROS production, which leads to lipid peroxidation, DNA damage, and ubiquitination. ROS induced by MPs also cause the triggering of stress related signaling pathways in the cells, which ultimately start the stimulation of transcription factors (e.g., AP-1 NFkB, NrF2), and subsequently generate gene expression that could end up initiating different biological responses, resulting in the stimulation of one or more signaling pathways leading to apoptosis and autophagy, as indicated by our results. All of these processes form a complexly inter-connected cascade in which the activation of one process triggers another [99].

Furthermore, lysosomal membrane stability of the hemocytes was reduced in both fishes after PP-MP exposure, with the difference that in *P. fluviatilis*, the maximal reduction observed was already achieved at the low dose, providing an indication that hemocytes are more easily affected by the exposure to PP-MPs. It is likely that hemocytes, as immune cells, internalize PP-MPs to a greater extent by phagocytosis, a function of the hemocytes intended to remove and kill hostile cells [100].

The sensitivity of the two fish species studied differed, according to the parameters measured. *D. rerio* was more vulnerable regarding DNA damage, Bax/Bcl2, and caspases compared to *P. fluviatilis* after their exposure to both concentrations of PP-MPs. On the other hand, *P. fluviatilis* responded with more sensitivity than *D. rerio* when lysosomal stability and autophagy indicator LC3/II were studied. Moreover, the tissue specificity in both fish species was not clear since it was dependent on the biochemical parameter examined.

The metabolome of both tissues of *P. fluviatilis* was altered in both treatments. Generally, the metabolome of the liver was more affected than that of the gills, particularly with regard to metabolites related to the metabolism of energy, neurotransmitters, and nucleic acids. In both tissues, considerable reductions were observed in the levels of metabolites related to nervous function; tryptophan, nicotinic acid, and γ-aminobutyric acid exhibited the greatest decrease among all metabolites in all treatments in comparison to the control, an interesting observation since tryptophan is the metabolic precursor of nicotinic acid, which is related to maintaining the health of the nervous system. In contrast, in the gills at 10 mg/g PP-MPs, most amino acid levels increased in relation to the control, probably as a result of protein breakdown caused by ubiquitination and autophagy.

The results of the present study are critical for evaluating the impacts of MPs on aquatic ecological security and sustainability. They also confirm the previous data of our research group on the validity and reliability of the use of the specific group of biomarkers for biomonitoring experiments in aquatic environments not only against PP-MPs but also against PS-MPs and PE-MPs as well as against nanoparticles.

## Figures and Tables

**Table 1:** Fold changes (%) of metabolites between the exposed and control in the perch gills and liver (for 1 mg/g and 10 mg/g MP particles in feed).

		Compound Name	Gills		Liver	
			1 mg/g	10 mg/g	1 mg/g	10 mg/g
Neurotransmitter		γ-Aminobutyric acid	−36	−22	−66	−41
Amino acids	Aromatic	Tryptophan	−95	−96	−58	−52
		Tyrosine	−42	294	387	2
		Phenylalanine	−37	206	67	−31
	BCAA	Leucine/Isoleucine	−9	213	29	−34
		Valine	−12	121	36	−33
	Hydrophobic	Proline	−11	144	22	−24
		Alanine	−22	71	−35	−52
		Serine	−55	−10	92	−39
	Acidic	Glutamic acid	30	0	139	−23
		Aspartic acid	−23	−28	6	−30
Energy metabolism		Lactic acid	−33	−33	60	−52
		Creatine	28	95	−32	−10
		Citrulline	−42	142	31	−28
		Carnitine	3	412	−82	−29
		Acetylcarnitine	−20	−16	−85	10
		Glucose/Galactose	21	−0	−95	−23
		Fumaric acid	22	−52	−41	−50
		Nicotinic acid	−72	−84	−91	−58
		Nicotinamide	−6	−37	−57	−2
Nucleic acid metabolism		Ribose	−9	−29	−50	−92
		Adenine	−19	7	−52	−60
		Adenosine	−16	213	−36	−57
		Guanine	6	−31	−88	−88
		Cytosine	5	−38	−1	−79
		Uracil	218	−18	52	−19
		Uridine	60	42	0	−28
		Hypoxanthine	31	−13	−21	−26
		Choline	24	−31	77	1
		Betaine	37	120	43	−8

## Data Availability

Data are available from the corresponding author upon reasonable request.

## Supplementary Materials

The following supporting information can be downloaded at: https://www.mdpi.com/article/10.3390/ijms232213878/s1.
