# Supplementary material for: Dose-Dependent Cytotoxicity of Polypropylene Microplastics (PP-MPs) in Two Freshwater Fishes
Source: Int J Mol Sci. 2022 Nov 10;23(22):13878. doi: 10.3390/ijms232213878 (PMC9692651; doi:10.3390/ijms232213878)
Supplement: Supplementary file 1 [file ijms-23-13878-s001.zip › ijms-2013001-supplementary.pdf]

Supplementary Materials

FTIR is a spectroscopic technique that exhibits strong intensity signals at 2920 and 2950 cm^{-1} corresponding to polypropylene (PP), assigned to asymmetric stretching of CH_2 and CH_3 respectively. Figure S1 exhibits the spectra of the control zebrafish (a) and perch (b) liver, while the control FTIR spectra of gills for the two fishes are exhibited in Figure S1 (c) and (d) respectively.

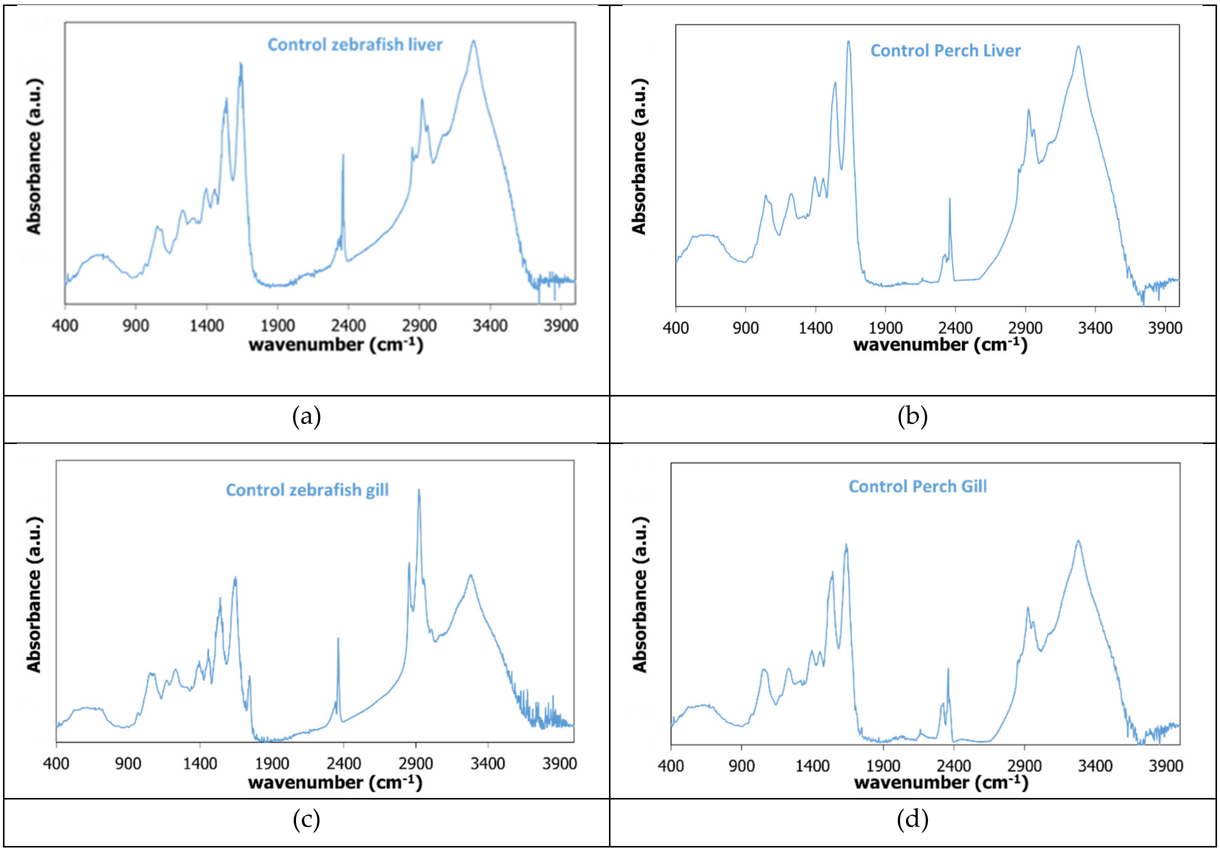

Figure S1. FTIR spectrum of the control (a) zebrafish liver, (b) perch liver, (c) zebrafish gill and (d) perch gill.

The FTIR spectra of the exposed gills and liver samples (Figure S1 in the text) exhibit similar peaks to the control samples, which are mainly attributed to proteins; between 900 and 1300 cm^{-1} are phosphates mainly associated with RNA and DNA related nucleic acids, in the 1300 and 1800 cm^{-1} region are protein (Amide I, II) bonds and in the 2700-3900 cm^{-1} wavenumbers are peaks related to N-H stretching vibration of proteins and these peaks are met in all gills and liver samples of both perch and zebrafish.

From SEM micrographs (Figure S2) it was found that the prepared PP microparticles are spherical in shape with particle sizes diameters ranged from 3-7 μm.

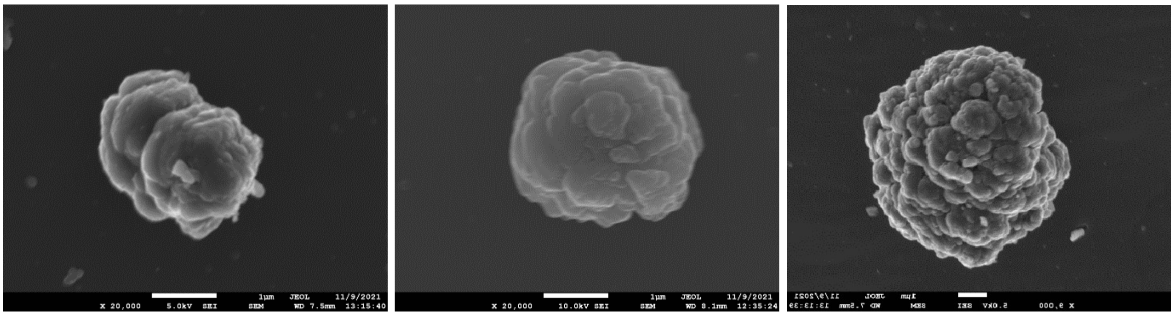

Figure S2: SEM micrographs of prepares PP microplastics.
